# Unplanned antiretroviral treatment interruptions in southern Africa: how should we be managing these?

**DOI:** 10.1186/1744-8603-6-4

**Published:** 2010-03-31

**Authors:** Nina Veenstra, Alan Whiteside, David Lalloo, Andrew Gibbs

**Affiliations:** 1 Health Economics and HIV/AIDS Research Division, Westville Campus, University of KwaZulu-Natal, Durban, South Africa; 2 Liverpool School of Tropical Medicine, Pembroke Place, Liverpool, UK

## Abstract

Adherence to antiretroviral therapy is essential for maximising individual treatment outcomes and preventing the development of drug resistance. It is, however, frequently compromised due to predictable, but adverse, scenarios in the countries most severely affected by HIV/AIDS. This paper looks at lessons from three specific crises in southern Africa: the 2008 floods in Mozambique, the ongoing political and economic crisis in Zimbabwe, and the 2007 public sector strike in South Africa. It considers how these crises impacted on the delivery of antiretroviral therapy and looks at some of the strategies employed to mitigate any adverse effects. Based on this it makes recommendations for keeping patients on treatment and limiting the development of drug resistance where treatment interruptions are inevitable.

## Review

Antiretroviral therapy (ART) adherence is compromised under some situations in countries most heavily affected by HIV/AIDS. A frequently cited meta-analysis affirmed that patients in sub-Saharan Africa (sSA) record adherence levels as good as those documented in the rich world [1]. However, unfavourable contexts limit an individual's control over their own treatment.

Many early ART programmes which were the subject of adherence studies in sSA captured a set of circumstances that cannot consistently be maintained in the longer-term as treatment is scaled up. In particular, inconsistent drug supplies have been shown to be an important factor influencing adherence [see for example [2-5]]. Both direct and indirect costs, a function of the broader socioeconomic environment, also feature prominently in relevant research [see for example [6-10]].

Any threats to ART adherence need to be taken seriously if we are to optimise treatment outcomes for individuals. Robust evidence exists on the effect of adherence on viral load [11-15], the immune system [16-18], and clinical prognosis [16,19-23]. Most importantly perhaps, the increased risks of illness and death amongst those that are poorly adherent are undeniable. A recent study in South Africa concluded those patients claiming less than 80% of their prescription refills were over three times more likely to die than those claiming more than 80% [21].

The development of drug resistance due to poor adherence goes beyond the individual level. It becomes a public health issue when drug resistant viral strains are transmitted and this has been a major concern amongst governments and health agencies. As treatment was introduced in sSA, we were warned of a situation of 'antiretroviral anarchy' where the rapid emergence and transmission of resistant viral strains would ultimately limit treatment options [24]. Fortunately, transmitted resistance in countries in sSA currently scaling up ART programmes is still less than 5%, but needs to be monitored closely [25]; increasing levels of resistance are inevitable as treatment coverage expands. With non-nucleoside reverse transcriptase inhibitor (NNRTI)-regimens in particular (used most commonly in sSA), drug resistance can develop after unplanned treatment interruptions of just a few days [26,27].

This article looks at ART adherence concerns arising from three specific crises in the southern African region, based on a literature review of reports and papers in the public domain. It considers the impact of these crises on adherence and explores strategies to try and keep patients on treatment, or to interrupt their treatment safely. It uses this as a basis for making suggestions as to how ART interruptions arising from various scenarios might be avoided and managed in future.

### What types of situations compromise ART adherence and how does this happen?

There are a huge range of different crises that can potentially undermine ART treatment in southern Africa and the broader region. These crises are of different natures, various durations (short term vs long term) and vary in geographical extent (localised vs widespread). They also manifest in different ways. This paper looks at problems with health system functioning and ART delivery during: 1) the 2008 floods in Mozambique, 2) the ongoing political and economic crisis in Zimbabwe, and 3) the 2007 public sector strike in South Africa (see Table 1). While each crisis is irrefutably unique in many ways, we have used some of the obviously classifiable features to frame the recommendations.

**Table 1:** Three crises in southern Africa that have impacted on ART adherence

Nature of crisis	Case study	Major effect of crisis	Duration of crisis	Extent of crisis
1. Natural disasters	2008 floods in Mozambique	Migration, damage to health system infrastructure	Short to medium term	Localised

2. Political and economic failure	Ongoing situation in Zimbabwe	Poverty, migration, health system collapse	Long term	Widespread

3. Health service or system failure	2007 public sector strike in South Africa	Poor access to health services	Short term (but similar nature crises can be longer term)	Widespread (but similar nature crises can be localised)

In the case of natural disasters (and particularly floods), health concerns are often dominated by sanitary problems and overcrowding in temporary camps, which increase the risks of diarrhoeal diseases, cholera, measles, and malaria [28]. In Mozambique reports suggested that 72 people died of cholera and an equal number of other waterborne diseases, a number far greater than the dozen or so that died as a result of rising flood waters [29]. The immediate need to intervene with safe access to clean water meant that other health problems were eclipsed. Some longer term risks were however identified, including: 1) poor access to food supplies, potentially resulting in malnutrition, and 2) poor access to health care, potentially resulting in worse maternal and child health outcomes and inadequate management of both acute and chronic diseases [30].

Situations that results in the displacement of large numbers of people means those on ART may not be able to access the medication they require. In Mozambique, fleeting mention was made in a few reports of the poor access to health facilities in resettlement areas, and the fact that health posts (providing very basic services) in these camps were slow to open due to a lack of drugs [31,32]. Medecins Sans Frontiers reported in late January 2008 that 60 patients in Mutarara (Tete province) on HIV and TB treatment were missing and had not come to the hospital to collect their monthly medication [33]. In this instance teams were sent out to find them, but public health services do not generally have the capacity to respond in this way.

Political and economic failure, which has widespread implications for the health system, is harder to manage and control for. Health care provision can become more difficult due to the lack of drugs and medical supplies, as well as insufficient numbers of health workers. In Zimbabwe this resulted in the closure of some of the largest hospitals in late 2008 [34]. Even where drugs were free and health services available, patients struggled to access them due to obstacles such as high fuel and transport costs [35]. Under such circumstances patients can't afford all the basic necessities and are forced to migrate, with deleterious consequences for treatment.

How do health workers and ART patients respond to such a chaotic situation and are these responses appropriate? In Zimbabwe there were reports of ART patients missing drug doses, sharing drugs, selling their drugs and changing regimens to try and cope with inadequate drug supplies and poor economic circumstances, so fuelling concerns of a drug-resistant HIV epidemic [34,36,37]. On a more positive note, aid agencies and donors were aware of the need to keep patients on treatment and played an important role in ensuring the provision of drug supplies for the ART programme.

The category of health system or service failure is all encompassing as it can manifest in many ways, with common examples including health worker strikes or drug stock-outs. Such problems are often symptomatic of broader political and/or economic collapse, although here we concern ourselves primarily with shorter term failures that are more confined to the health sector. These generally result in patients not accessing necessary health services for a limited period of time, as was the case during the month-long South African public sector strike in 2007. In this instance there were mixed reports over the extent of the disruption, but a significant number of patients with HIV/AIDS and TB could not obtain their treatment because of clinic closures [38,39].

As the duration of the public sector strike was short term, patients with buffer drug stocks would have possibly been able to weather the crisis with little disruption to treatment. However, in most cases patients only obtain their drugs on a month by month basis and so would have had little reserve. The Southern African HIV Clinicians Society took the initiative of issuing a press statement on how treatment interruptions should be dealt with. This essentially advised patients to seek help from other sources (either private pharmacies or another hospital/clinic) and to stop all drugs simultaneously if need be, without decreasing the dose to make treatment last longer [40]. Unfortunately, due to its late release in media that few have access to and because it was written in English, it is unlikely that this information would have reached many of the ART patients who needed it.

### What strategies should be considered to keep ART patients adherent on treatment?

The two key messages from this review are: 1) we need to plan for and manage various crises impacting on ART delivery; and 2) there are a number of strategies that have been employed and should be evaluated. Unfortunately, most of what has been tried to date has been 'too little, too late' and there has been almost no indication as to its effectiveness. Nonetheless, there are conclusions and recommendations arising from the review that provide a starting point.

Management strategies will vary according to the duration of the crisis. Many shorter term crises like those reviewed in this paper can be anticipated, indicating a need for sound planning and preparedness. Patients could be given extra drug supplies to tide them over a potentially difficult time. Anecdotal evidence suggests that this strategy is used to help patients cope with short periods of planned migration. It does, however, come with the concern that relaxing controls on ART might result in drugs being shared or sold on the black market. It therefore has to be managed carefully.

Longer term crises, in contrast, cannot be planned for so easily because of the chronic erosion to the health system and the time taken to potentially restore services. In these cases, close monitoring and proactive management is needed to alleviate the effects of the crisis on patient treatment, care and support. Health information systems are central; needing to be sensitive enough to alert managers to change (such as when fewer patients start presenting for follow up or drug shortages are imminent) and detailed enough to give a fairly complete picture of what is happening. Our observation that so little information exists on the impact of crises on ART programmes, is testament to how poor such systems currently are. It is therefore no surprise that management of disruptions to ART delivery is largely ineffective. Looking beyond the crisis itself, a carefully designed recovery plan will assist in restoring health system functioning as quickly as possible.

Just as shorter term crises will be easier to weather than longer term crisis, so too will localised disruptions compared to widespread upheaval. Careful management and some reorganisation of services may suffice with localised disruptions. There was some evidence of this happening in Mozambique, where new health posts were set up and community workers used to search for patients [31]. Such strategies do, however, have to be implemented quickly and efficiently if they are to ensure patient adherence to medication. The use of mobile phones to keep contact with patients could be explored.

Widespread upheaval on the other hand, requires an increased involvement and co-ordination of health sector partners, be they private, donor, NGO or faith based. There were indications from Zimbabwe that such partners played an important role in ensuring the continuation of ART drug supplies, despite operating in a hostile environment where drug delivery and distribution were problematic [34]. In South Africa private health care providers took care of many Intensive Care Unit (ICU) patients during the strike [41]. In a country like this, where the private health sector is large, there is scope for exploring partnerships that could benefit a wider range of patients in times of crisis.

Crises that are both widespread and longer term, like that in Zimbabwe, often require a particularly holistic approach to support patients on ART. This is because the socio-economic circumstances of households become compromised, meaning that simply ensuring ongoing service delivery is not sufficient to maintain ART adherence. In such cases extra assistance may be required from a range of partners to manage the indirect costs of seeking care, such as transport costs, and to avoid a situation where people are having to trade-off basic necessities.

For all types of crisis, whether long or short term, widespread or localised, patient information systems which facilitate tracking and enable patients to collect their drugs from multiple locations would help tremendously. This is because migration is often a common outcome of a crisis. Even in South Africa, where patients did not migrate during the strike, they were advised to try and source their medication from other health facilities or private pharmacies [40]. However, patients do not carry any record of their medication on them and so were told to take their pills along instead for the doctor or pharmacist to identify, which obviously is not ideal.

Lastly, the impact of deficient ART adherence during crisis situations could be lessened through attention to the basics - patient adherence counselling and health worker training. If adherence counselling includes discussion on what to do in case of being unable to access drugs, then it should be possible to avoid some of the inappropriate coping strategies (such as sharing drugs or reducing drug doses) reported in Zimbabwe. Clinicians properly trained in managing treatment interruptions could stagger the time at which different drug classes are stopped during drug stock-outs to avoid the development of NNRTI resistance in particular. Issuing such guidance during a crisis, as was done in South Africa [40], might supplement such measures but is unlikely to be effective on its own.

### Who takes responsibility?

There is no doubt that widespread, long term crises, particularly those involving state failure and political violence (such as in Zimbabwe), are the greatest threat to ART adherence. In such situations there is an urgent need to consider the question of responsibility. If government failure is making it difficult for people to obtain the treatment they need and NGOs within the country are struggling to operate in a hostile environment, should there not be some international responsibility for people living with HIV/AIDS?

In addressing the situation of people living with AIDS in Zimbabwe, there is scope to consider how the emerging 'Responsibility to Protect' (RtoP) doctrine could be applied [42]. The RtoP considers that 'sovereign states bear the responsibility to protect their citizens from avoidable catastrophe, but that when they are unwilling or unable to do so, that responsibility must be borne by the broader community'[43]. It therefore sets new norms for international security and human rights.

In the case of Zimbabwe, many patients on ART struggled to access their treatment not only within the country's borders, but because they were forced to migrate to neighbouring countries, especially South Africa. Undocumented migrants in South Africa have a constitutional right to access ART, but many remain unable to do so [44]. Furthermore, one can argue that these 'migrants' are in fact refugees, in which case the role of the United Nations High Commissioner for Refugees (UNHCR) in supporting access and adherence to ART comes into question. The UNHCR has pledged to keep HIV/AIDS as priority [45], however it is unclear what is being done in this regard.

## Conclusions

In conclusion, ART programmes in sSA will continue to be challenged by a range of different crises. What we have seen to date is that the need to prioritise often results in immediate concerns arising from such crises eclipsing those with longer-term consequences. HIV/AIDS is by nature a long-term disease and failure to maintain treatment coverage and adherence will not immediately result in large numbers of deaths. Unfortunately this does not mean that the consequences will be less severe - only that they will play out for years to come. Responding strategically to the various threats posed by a crisis calls for careful consideration and co-ordination, rather than a knee-jerk response. Ultimately, how we plan for and manage such crises could strongly influence treatment outcomes in the years and decades to come.

## Competing interests

The authors declare that they have no competing interests.

## Authors' contributions

NV contributed to the design of the study, undertook the literature review and was primarily responsible for drafting the manuscript. AW conceptualised the design of the study and helped with revising the manuscript for publication. DL conceptualised the design of the study and helped with revising the manuscript for publication. AG helped with revising the manuscript for publication. All authors read and approved the final manuscript.
